# A Pilot Comparative Study of Multi‐Contrast Dental MRI and CBCT: Clinical Correlates of Disease Status and Inflammatory Burden in Periodontitis

**DOI:** 10.1111/jcpe.70151

**Published:** 2026-06-17

**Authors:** Nouha Tekiki, Arne Lauer, Katharina Hartung, Marietta Kirchner, Luisa Schulte, Hao Li, Alexander Juerchott, Meysam Sohani, Christopher Herpel, Maurice Ruetters, Mathias Nittka, Sabine Heiland, Martin Bendszus, Tim Hilgenfeld

**Affiliations:** ^1^ Department of Neuroradiology Heidelberg University Hospital Heidelberg Germany; ^2^ Institute of Medical Biometry, Heidelberg University Hospital Heidelberg Germany; ^3^ Private Practice Schwalmtal Germany; ^4^ Department of Prosthodontics Heidelberg University Hospital Heidelberg Germany; ^5^ Section of Periodontology, Department of Operative Dentistry Heidelberg University Hospital Heidelberg Germany; ^6^ Research & Clinical Translation, Magnetic Resonance, Siemens Healthineers AG Erlangen Germany

**Keywords:** cone‐beam computed tomography, dental MRI, magnetic resonance imaging, periodontal inflamed surface area, periodontitis, periodontium, reliability

## Abstract

**Aim:**

To evaluate the reliability of multi‐contrast dental MRI (dMRI) for periodontal lesion assessment in comparison to cone‐beam computed tomography (CBCT). Associations between imaging findings and clinical indicators of periodontal inflammation (i.e., bleeding on probing [BOP], periodontal probing depth [PPD] and periodontal inflamed surface area [PISA]) were analysed to explore the potential of dMRI to reflect disease status and inflammatory burden.

**Materials and Methods:**

In this prospective study, patients with severe periodontitis underwent clinical examination, CBCT and 3T dMRI. Site‐level detectability of periodontal osseous defects and/or marrow signal changes at the six standard clinical probing sites per tooth, as well as tooth‐level lesion volumetry, were evaluated across CBCT, T1‐weighted (T1W), contrast‐enhanced T1W and T2‐weighted (T2W). Reliability (κ, ICC) and clinical‐imaging associations (Kruskal–Wallis and mixed‐effect models) were analysed.

**Results:**

Nineteen patients with severe periodontitis stages III and IV were included. Reliability of dMRI matched CBCT (site‐level *κ* = 0.631–0.771; tooth‐level ICC = 0.832–0.973). Increasing PPD and BOP raised lesion detectability and volumes across all modalities, with dMRI being consistently more sensitive than CBCT. T1W MRI showed lesions and bone loss similar to CBCT and was unaffected by BOP (*p* = 0.132) or PPD (*p* = 0.252). T2W MRI identified most lesions regardless of PPD and reflected high correlations with BOP (*p* < 0.05), PPD (*p* < 0.001) and PISA (*p* < 0.001).

**Conclusions:**

Reliability of dMRI for lesion identification and quantification was comparable to that of CBCT. The presence of T2W marrow oedema at sites with a CBCT‐normal periodontal ligament space and no radiographically detectable bone loss may represent a promising imaging marker for early periodontal disease recognition and assessment of inflammatory burden.

## 1 Introduction

Severe periodontitis affects over one billion people worldwide and is the sixth most prevalent disease, leading to tooth loss, compromised oral function and quality of life (Nascimento et al. 2024; Wu et al. 2025; Tonetti et al. 2017). It is also increasingly linked to systemic conditions such as cardiovascular, cardiometabolic and autoimmune disorders (Tonetti et al. 2017; Elebyary et al. 2024). Current periodontal diagnosis relies on clinical measurements obtained by inserting a periodontal probe into the gingival sulcus to assess periodontal probing depth (PPD), clinical attachment loss (CAL) and bleeding on probing (BOP), together with radiographic alveolar bone loss. PPD, CAL and radiographic bone loss reflect predominantly the structural state of the periodontium at the time of examination, and are limited in their ability to detect present ongoing inflammation or sites at risk of future tissue breakdown (Elebyary et al. 2024; Landzberg et al. 2015).

Clinically, inflammatory status is inferred from BOP and PPD (Chapple et al. 2018; Tonetti et al. 2018; Zimmermann et al. 2015). However, deep periodontal pockets do not necessarily represent current inflammation and may remain stable and uninflamed over extended periods (Landzberg et al. 2015; Lang and Bartold 2018). Likewise, bleeding does not reliably indicate ongoing tissue destruction, with a sensitivity of 29% and positive predictive value of only 6% for future progression (Landzberg et al. 2015; Lang et al. 1990). Periodontal inflamed surface area (PISA), a novel inflammatory marker, represents a continuous measure of the amount of inflamed periodontal tissue (Nesse et al. 2008; Park et al. 2017). Because it weights bleeding by pocket depth, PISA is more sensitive than BOP% for detecting progression of inflamed periodontal tissue in longitudinal studies (Nomura, Morozumi, Saito, et al. 2021). Moreover, it has been linked to C‐reactive protein, serum interleukins and systemic diseases (Nesse et al. 2009; Nomura, Morozumi, Numabe, et al. 2021; Matsuda et al. 2024).

As for radiographic assessment, two‐dimensional (2D) imaging underestimates initial bone loss because of its inability to detect early demineralisation and overestimates severe bone loss due to superimposition and projection errors (Discepoli et al. 2025). Cone‐beam computed tomography (CBCT) provides a more reliable assessment of intrabony defects and furcation involvement and true volumetric information (Discepoli et al. 2025; Walter et al. 2009; Qiao et al. 2014) but remains a static modality that visualises only established bone loss and cannot depict inflammatory infiltrate, oedema or marrow changes preceding radiographically apparent destruction. Its associated radiation exposure also limits its suitability for screening or repeated longitudinal monitoring (Jacobs et al. 2024; Mahesh et al. 2022). Combining all the evidence, a recent consensus concluded that no genomic, host‐derived or microbial marker can accurately predict disease progression or treatment success (Herrera et al. 2025).

Magnetic resonance imaging (MRI) is uniquely sensitive to soft‐tissue composition, fluid content and bone marrow changes and seems promising for periodontal diagnostics (Herrera et al. 2025). MRI was initially applied to visualise and evaluate periodontal and peri‐implant soft tissues, but has since expanded to the assessment of periodontal bone involvement and intraosseous inflammatory changes (Yeung et al. 2024). Previous dental MRI (dMRI) studies have shown that fat‐suppressed (fs) T1‐weighted (T1W) and contrast‐enhanced (T1WC) contrasts reliably detect furcation involvement (Juerchott et al. 2020a, 2020b), and fs T2‐weighted (T2W) contrasts visualise marrow oedema beyond bone loss detectable on orthopantomography (OPT) (Probst et al. 2021). Linear and volumetric T2W oedema and volumetric T1WC marrow signal changes have been correlated to BOP and PPD (Probst et al. 2021; Schwarting et al. 2024; Lauer et al. 2025). However, no study to date has comprehensively assessed all major dMRI weightings directly compared to CBCT and examined how each modality or sequence relates to clinical inflammatory indicators across the full dentition. This study also incorporated PISA, a previously unused metric, to bridge the gap between linear clinical measures and 3D volumetric imaging, serving as a validated intermediate square measure of inflamed periodontal surface area, and strengthening interpretation of imaging‐derived inflammatory changes in the context of clinical findings.

This study aimed to determine the reliability of dMRI for assessing inflammatory periodontal imaging findings at both site and tooth levels by comparing T1‐, contrast‐enhanced T1‐ and T2‐weighted sequences with CBCT to define which components of periodontal disease each imaging modality or sequence captures, and to identify which dMRI sequence best reflects periodontal inflammatory status and burden.

## 2 Methods

### 2.1 Ethics and Patients

This study was approved by the ethics committee of the University of Heidelberg (S‐452/2010) and performed in accordance with the Declaration of Helsinki. Written and informed consent was obtained from each patient. Full‐mouth periodontal examinations were performed by two procedurally calibrated dentists. PPD and gingival recession (REC) were measured to the nearest millimetre at six sites per tooth (mesio‐buccal, buccal, disto‐buccal, disto‐palatal, palatal and mesio‐palatal) using a periodontal probe (HS‐Parodontometer Figur CP15; Henry Schein Inc.). CAL was measured indirectly as the sum of PPD and REC. BOP was recorded at each site after a single probing. Periodontitis was diagnosed according to the 2017 World Workshop criteria (Tonetti et al. 2018). PPD, BOP, CAL and/or REC were used to calculate PISA using the formula by Nesse et al., excluding third molars (Nesse et al. 2008). See Appendix for additional information regarding study participants.

### 2.2 CBCT and dMRI Examinations

CBCT images were acquired under standard clinical indications, that is, including assessment of advanced furcation involvement and preoperative implant planning. MRI imaging was performed solely as part of the research protocol.

CBCT images were acquired using a 3D Accuitomo 170 system (J Morita; Kyoto, Japan) with operating parameters as follows: cylindrical volume range: 4 × 4–8 × 8 cm; tube voltage: 90 kV; tube current: 5 mA; rotation: 360°; scanning time: 17 s; isotropic voxel size: 0.16 mm.

All dMRI images were acquired on the same day as CBCT using a 3‐T MRI system (MAGNETOM Trio; Siemens Healthineers, Forchheim, Germany) and a dedicated 15‐channel dental coil (Mandibula, Noras MRI products GmbH, Hoechberg, Germany). Table S1 summarises technical parameters of the acquired sequences: A 3D fs T2W MSVAT‐SPACE research sequence (Li et al. 2011) and 3D T1W VIBE with Dixon‐based fs sequences before and after intravenous gadolinium administration. All sequences were acquired in < 20 min.

### 2.3 Image Processing and Periodontal Lesion Analysis

All CBCT and MRI datasets were anonymised and analysed using the 3D SLICER software (https://www.slicer.org; Version 5.6.1). Imaging assessments were performed blinded to clinical findings by independent examiners. All periodontal lesions—whether suprabony (horizontal), infrabony (vertical) and inter‐radicular osseous defects—were included in the assessment. Imaging presentation of periodontal lesion on CBCT and MRI is described in Appendix A.

### 2.4 Site‐Specific Analysis: Periodontal Lesion Detection

**FIGURE 1:**
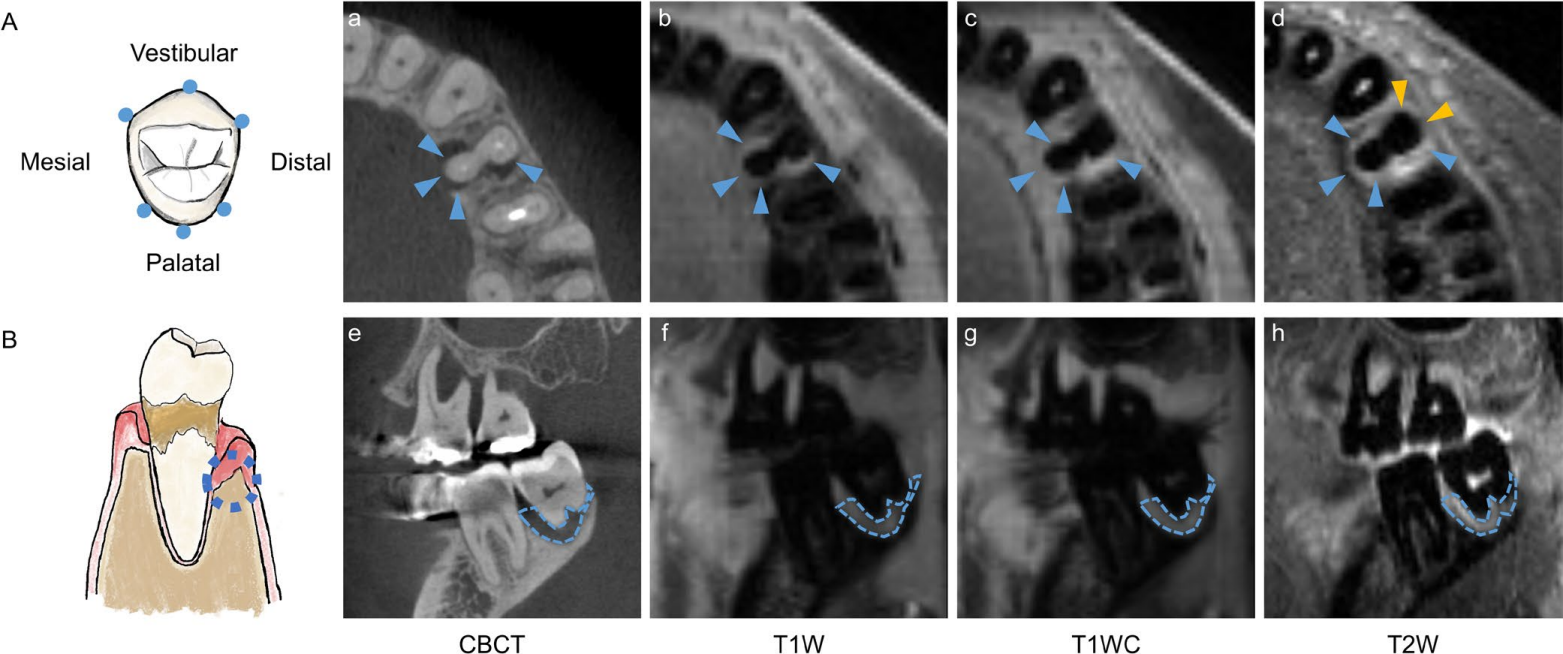
Site‐specific (A) and tooth‐specific (B) analyses of periodontal lesions on images of a 53‐year‐old female patient with severe periodontitis. Periodontal lesions (arrowheads) were screened for at six sites per tooth corresponding to standard clinical probing sites and recorded as present or absent (A: a, b, c, and d). Note the juxtaposition of areas exhibiting periodontitis‐associated bone loss in CBCT and MRI (coloured blue) and areas illustrating MRI signal alterations only (coloured yellow). For volumetric analysis, lesions were delineated voxel by voxel in each image and slice of each imaging modality (B: e, f, g and h). Imaging modalities include cone‐beam computed tomography (CBCT: a, e), T1‐weighted (T1W: b, f), contrast‐enhanced T1‐weighted (T1WC: c, g) and T2‐weighted (T2W: d, h) contrasts.

Lesions were visually assessed at six sites per tooth corresponding to the clinical probing sites in a blinded fashion. Each site was recorded as lesion present or absent (Figure 1A).

### 2.5 Tooth‐Specific Analysis: Periodontal Lesion Volumetrics

Lesion volumes were quantified at tooth level because of the confluent nature of lesions—which often affected multiple adjacent probing sites around a single tooth—also in a blinded fashion. Lesion boundaries for volumetric delineation were based on the visually identifiable margins of osseous defects (CBCT) and marrow signal alterations (MRI) (Figure 1B). See Appendix A for segmentation.

### 2.6 Reliability Analysis of Image Assessments

Among modalities, dMRI was evaluated first, before CBCT with a ≥ 8‐week interval, to avoid learning bias. Detailed description of the readers' expertise, calibration and reliability assessment are provided in Appendix A.

### 2.7 Clinical Findings and Periodontal Lesions on Imaging

PPD and BOP were first analysed separately to assess their individual associations with imaging expression of periodontal lesions, and subsequently analysed together to reflect their coexistence and interrelated behaviour in the clinical setting and to evaluate their combined association with imaging findings.

At the site level, the association between PPD and imaging‐based lesion detection was examined, categorising sites as negative (PPD = 0 mm), shallow (PPD ≤ 3 mm) and deep (PPD > 3 mm). The latter was defined at 4 mm PPD, as it represents a critical threshold for determining periodontal stability at non‐bleeding sites following successful periodontal therapy (Dietrich et al. 2019). Detection rates were compared across these categories. The rationale is that detectability likely increases with local morphological and inflammatory alterations, clinically reflected by deeper pockets.

At the tooth level, the association between BOP and lesion volumetrics was evaluated. Teeth were separated by the number of BOP sites (BOP^0^, BOP^1+^, BOP^2+^, BOP^3+^, BOP^4+^, BOP^5+^ and BOP^6+^). Analyses were restricted to lesions detected across all imaging modalities to allow valid comparisons. Absolute and volume differences between dMRI sequences and CBCT were assessed in relation to BOP severity, under the rationale that more bleeding sites reflect more extensive inflammatory involvement, manifesting as wider oedema or signal abnormalities on dMRI.

Finally, the overall relationship between periodontal inflammatory status and imaging findings was assessed using average PPD and number of BOP sites. PISA was evaluated separately to avoid multicollinearity. These analyses aimed to characterise the additional lesion extent on dMRI (marrow oedema) relative to CBCT and explore whether specific MRI sequences correspond more strongly with periodontal inflammatory status and burden.

### 2.8 Statistical Analysis

Statistical analyses were performed using IBM SPSS Statistics Version 29 (IBM Corp, Armonk, NY), MATLAB R2024b or SAS version 9.4 (SAS Institute Inc). Intra‐ and inter‐rater reliability were evaluated by Cohen's kappa (*κ*) and intra‐class correlation coefficient (ICC) (Landis and Koch 1977a, 1977b). Normal distribution was assessed using the Shapiro–Wilk test. Volumes and differences between dMRI and CBCT were tested using the Kruskal–Wallis test with Bonferroni's correction. Exponential regression modelled additional dMRI extent versus the number of BOP sites. Linear mixed models (Kenward–Roger degrees of freedom) with a random effect for patient were used to assess the relationship between additional extent and clinical parameters, with CBCT volume as a covariate. Two‐tailed *p*‐values < 0.05 were considered statistically significant (For detailed information, see Appendix A).

## 3 Results

Twenty‐two patients were enrolled in the cohort during the study period and assessed for eligibility. Nineteen patients (10 women, 9 men; age range 31–70 years, mean age 53.4 ± 14.7 years) with severe periodontitis stage III (grade B/C: 6/3) and stage IV (grade B/C: 3/7) were included. Across all modalities, nearly 64,000 images were analysed. Exclusion due to metal artefacts was 8.3% and 7.0% for site‐ and tooth‐level analyses. In total, 1986 periodontal sites and 342 teeth, with available clinical and imaging data, were included.

### 3.1 Diagnostic Reliability of Site‐Specific and Tooth‐Specific Analyses

CBCT and dMRI sequences (T1W, T1WC and T2W) all showed substantial to almost perfect intra‐/inter‐rater agreement for site‐specific lesion detection (*κ* range = 0.703–0.771/0.631–0.736) and tooth‐specific lesion volumetrics (ICC range = 0.885–0.936/0.832–0.973), with no systematic differences between CBCT and dMRI (Table S2).

### 3.2 Association Between PPD and Lesion Detection

Site distribution and lesion detectability are presented in Table 1 and Appendix B.

**TABLE 1:** Periodontal lesion detection at single sites according to probing pocket depth.

		Probing pocket depth (PPD)			Relative increase/decrease lesion detection (%)	
		Negative PPD (*n* = 1168)	Shallow PPD (*n* = 752)	Deep PPD (*n* = 66)	Negative to shallow PPD	Negative to deep PPD
Lesion detection						
CBCT, *n* (%)		593 (50.8)	390 (51.9)	55 (83.3)	+2.1	+64.1
T1W, *n* (%)		677 (58.0)	427 (56.8)	59 (89.4)	−2.0	+54.2
T1W compared to CBCT, (%)		+14.2	+9.5	+7.3		
T1WC, *n* (%)		741 (63.4)	461 (61.3)	60 (90.9)	−3.4	+43.3
T1WC compared to CBCT (%)		+25.0	+18.2	+9.1		
T2W, *n* (%)		958 (82.0)	571 (75.9)	63 (95.5)	−7.4	+16.4
T2W compared to CBCT (%)		+61.6	+46.4	+14.5		

Abbreviations: CBCT, cone‐beam computed tomography; PPD, probing pocket depth; T1WC, contrast‐enhanced T1‐weighted; T1W, T1‐weighted; T2W, T2‐weighted.

Lesion detection increased with greater PPD across all modalities. Between negative and shallow PPD, detection rates were nearly identical for CBCT (50.8%, 51.9%), T1W (58.0%, 56.8%) and T1WC (63.4%, 61.3%). In deep PPD, detection increased markedly, reaching 83.3%, 89.4% and 90.9% for CBCT, T1W and T1WC, respectively. In contrast to CBCT and the two T1‐weightings, lesion detectability on T2W was consistently high across all PPD groups (82.0%, 75.9% and 95.5%), with a smaller relative increase of +16.4% from negative to deep PPD.

Regardless of PPD, all MRI sequences detected more lesions than CBCT. For T1W and T1WC, the additional detection relative to CBCT decreased as PPD increased (T1W: 14.2%, 9.5% and 7.3%; T1WC: 25.0%, 18.2% and 9.1% in negative, shallow and deep PPD). Similarly, T2W exceeded CBCT; however, compared to T1‐weightings, its additional detection declined more steeply as PPD increased (61.6%, 46.4% and 14.5% in negative, shallow and deep PPD).

A supplementary analysis using combined PPD categories (PPD ≤ 3 mm vs. > 3 mm) is provided in Table S3.

### 3.3 Association Between BOP Sites and Lesion Volumes

Tooth distribution and lesion detectability are presented in Table 2 and Appendix B.

**TABLE 2:** Periodontal lesion volumes (median with IQR) at single teeth according to bleeding on probing.

Periodontal lesion volumes, median [IQR] [cc]	Bleeding on probing sites (BOP)								Relative (%) increase in lesion volume BOP^0^ to BOP^6+^
	BOP^0^‐teeth (*n* = 89)	BOP^1+^‐teeth (*n* = 70)	BOP^2+^‐teeth (*n* = 44)	BOP^3+^‐teeth (*n* = 56)	BOP^4+^‐teeth (*n* = 40)	BOP^5+^‐teeth (*n* = 10)	BOP^6+^‐teeth (*n* = 33)	*p* ^a^	
CBCT	0.016 [0.011–0.029]	0.018 [0.011–0.034]	0.035 [0.016–0.060]	0.023 [0.010–0.044]	0.031 [0.012–0.046]	0.032 [0.021–0.063]	0.027 [0.012–0.086]	0.219	+71.3
T1W	0.016 [0.011–0.035]	0.017 [0.012–0.038]	0.034 [0.018–0.067]	0.025 [0.013–0.045]	0.036 [0.014–0.047]	0.028 [0.022–0.071]	0.046 [0.016–0.101]	0.206	+182.1
T1WC	0.023 [0.014–0.050]	0.024 [0.015–0.050]	0.041 [0.022–0.091]	0.032 [0.015–0.062]	0.046 [0.026–0.061]	0.036 [0.020–0.057]	0.067 [0.028–0.132]	0.070	+191.3
T2W	0.046 [0.031–0.078]	0.054 [0.029–0.098]	0.094 [0.066–0.170]	0.080 [0.056–0.178]	0.069 [0.040–0.138]	0.087 [0.058–0.191]	0.175 [0.115–0.284]	**< 0.001** (***p* < 0.001** ^b^, ^c^)	+277.3

*Note:* Bold values indicate statistically signfificant *p*‐values (*p* < 0.05).

Abbreviations: BOP, bleeding on probing, CBCT, cone‐beam computed tomography; T1WC, contrast‐enhanced T1‐weighted; T1W, T1‐weighted; T2W, T2‐weighted.

^a^ Statistical difference via Kruskal–Wallis test. Post hoc pairwise comparison was performed using Bonferroni correction.

^b^ BOP^0^ vs. BOP^6+^.

^c^ BOP^1+^ vs. BOP^6+^.

Teeth with a higher number of BOP sites showed a gradual but nonlinear increase in lesion volumes across imaging (Table 2). Across the BOP groups, CBCT and T1W showed no significant differences in lesion volumes (*p* = 0.219 and *p* = 0.206; Kruskal–Wallis test), whereas T1WC showed a trend towards significance (*p* = 0.070). Only the T2W contrast demonstrated significantly larger volumes with increasing BOP (*p* < 0.001; Kruskal–Wallis test), in particular between BOP^0/1+^ and BOP^6+^ (*p* < 0.001, Bonferroni adjusted *p*‐value; Table S4).

**FIGURE 2:**
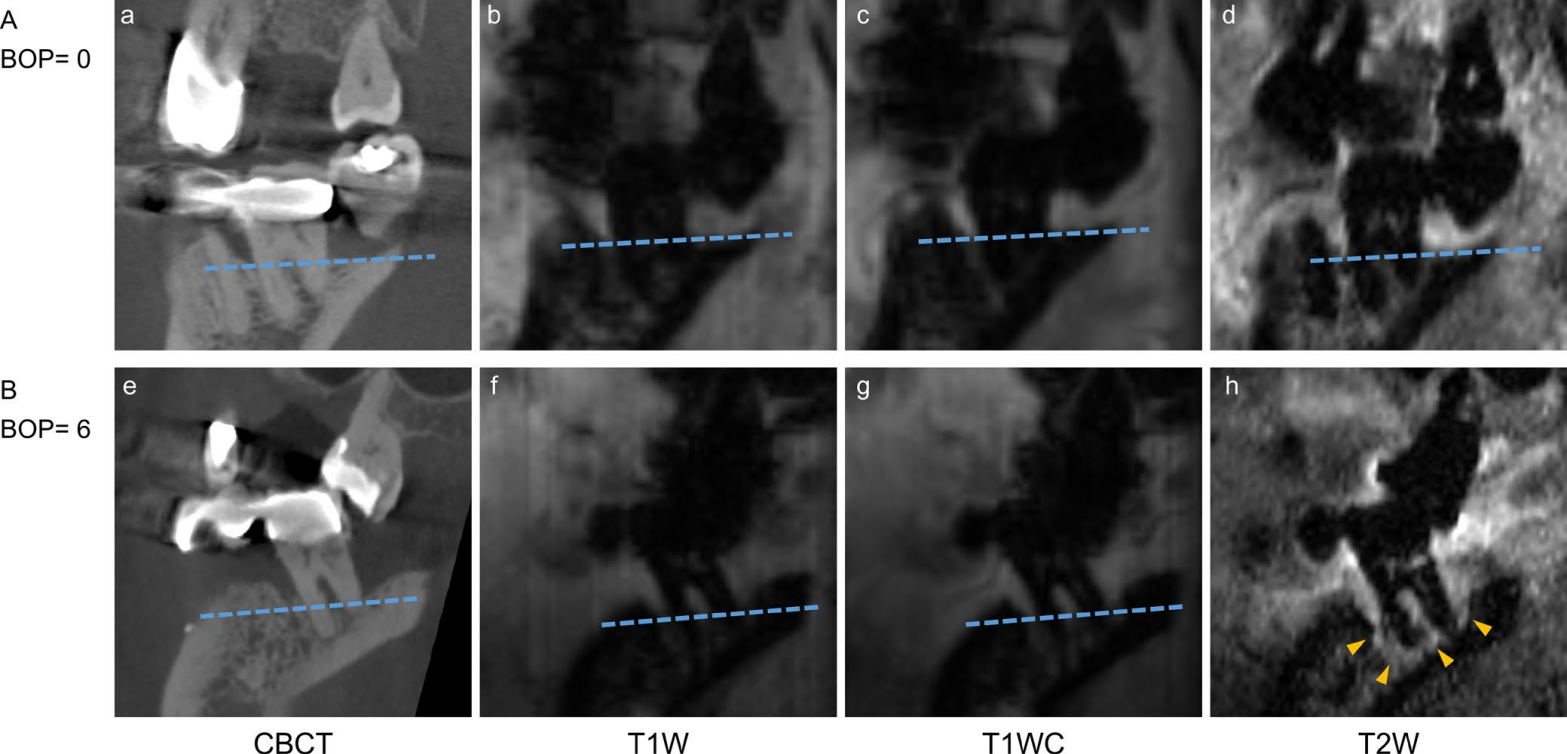
Images of teeth 36 and 37 with zero bleeding sites of a 61‐year‐old female patient (A) and tooth 47 with six bleeding sites of a 51‐year‐old male patient (B). Around teeth with no to minimal bleeding, periodontal lesions are expressed similarly across modalities, slightly larger in dMRI than in CBCT (dashed blue line) (A). The increasing severity of bleeding is expressed modestly and similarly in CBCT, T1W and T1WC, but evidently on T2W (B). T2W is showing an additional area with T2W‐only signal alteration beyond the bone loss (yellow arrowhead). Imaging modalities include cone‐beam computed tomography (CBCT: a, e), T1‐weighted (T1W: b, f), contrast‐enhanced T1‐weighted (T1WC: c, g) and T2‐weighted (T2W: d, h).

Regardless of BOP category, all dMRI sequences revealed larger lesion volumes than CBCT (Table 3). T1W versus CBCT showed negligible size differences (*p* = 0.531; Kruskal–Wallis test), which remained stable over BOP categories (median %: 12.2% at BOP^0^ to 14.1% at BOP^6+^). Larger, but still non‐significant differences were noted for T1WC (42.9% at both BOP^0^ and BOP^6+^; *p* = 0.128). In contrast, T2W revealed significantly larger lesions than CBCT at all BOP levels (*p* < 0.001). Volume difference to CBCT increased with growing number of bleeding sites (192.9% at BOP^0^ to 349.7% at BOP^6+^) (Figure 2).

**TABLE 3:** Periodontal lesion additional volume extent on dMRI relative to CBCT according to bleeding on probing.

Volumes being subtracted	Bleeding on probing sites (BOP)							
	BOP^0^‐teeth (*n* = 89)	BOP^1+^‐teeth (*n* = 70)	BOP^2+^‐teeth (*n* = 44)	BOP^3+^‐teeth (*n* = 56)	BOP^4+^‐teeth (*n* = 40)	BOP^5+^‐teeth (*n* = 10)	BOP^6+^‐teeth (*n* = 33)	*p* ^a^
T1W‐CBCT								
Median [IQR] [cc]	0.001 [−0.003–0.004]	0.003 [−0.003–0.007]	0.005[0.001–0.012]	0.002 [−0.001–0.010]	0.002 [−0.002–0.014]	0.003 [0.000–0.019]	0.005 [−0.001–0.019]	0.531
Median %	12.2	19.5	18.9	29.9	14.5	18.4	14.1	
T1WC‐CBCT								
Median [IQR] [cc]	0.006 [0.004–0.016]	0.007 [0.004–0.014]	0.008 [0.005–0.031]	0.008 [0.004–0.018]	0.011 [0.004–0.021]	0.001 [−0.008–0.014]	0.019 [0.006–0.049]	0.128
Median %	42.9	40.6	40.2	43.0	42.9	20.8	42.9	
T2W‐CBCT								
Median [IQR] [cc]	0.034 [0.012–0.056]	0.036 [0.010–0.067]	0.064 [0.036–0.114]	0.055 [0.027–0.099]	0.035 [0.021–0.119]	0.061 [0.044–0.130]	0.144 [0.069–0.284]	**< 0.001** (***p* < 0.001** ^b^, ^c^ ^,^ ^b^, ^[Link]^)
Median %	192.9	178.5	183.1	286.2	177.1	254.4	349.7	

*Note:* Bold values indicate statistically signfificant *p*‐values (*p* < 0.05).

Abbreviations: BOP, bleeding on probing; CBCT, cone‐beam computed tomography; T1WC, contrast‐enhanced T1‐weighted; T1W, T1‐weighted; T2W, T2‐weighted.

^a^ Statistical difference via Kruskal–Wallis test. Post hoc pairwise comparison was performed using Bonferroni correction.

^b^ BOP^0^ vs. BOP^6+^.

^c^ BOP^1+^ vs. BOP^6+^.

**FIGURE 3:**
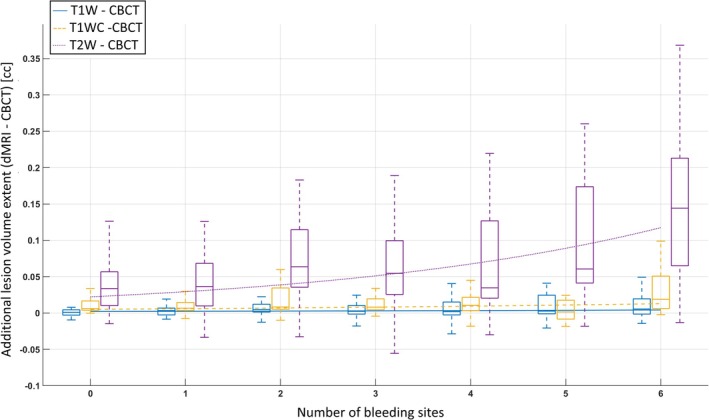
Association between bleeding severity and dMRI‐additional lesion extent relative to CBCT. Boxplots show the additional lesion extent for T1W (T1W‐CBCT, blue), T1WC (T1WC‐CBCT, yellow), and T2W (T2W‐CBCT, purple) across teeth grouped by the number of BOP sites (0 to 6). T1W and T1WC demonstrate minimal change in volume difference across BOP categories (*R* ^2^ = 0.20 and *R* ^2^ = 0.22), indicating limited responsiveness of these sequences to periodontal inflammatory indicators. In contrast, T2W shows a progressive and disproportionate rise in volume difference with increasing BOP, reflected by the steep exponential fit (*R* ^2^ = 0.62), indicating strong alignment with inflammatory indicators. Cone‐beam computed tomography (CBCT), T1‐weighted (T1W), contrast‐enhanced T1‐weighted (T1WC) and T2‐weighted (T2W).

Exponential regression models fitted to dMRI–CBCT volume difference as a function of BOP sites (Figure 3) identified weak, non‐significant associations for T1W–CBCT (*R* ^2^ = 0.20) and T1WC–CBCT (*R* ^2^ = 0.22). In contrast, T2W–CBCT demonstrated a strong, significant association with BOP (*R* ^2^ = 0.62).

### 3.4 Association Between Periodontal Inflammatory Status and Imaging Findings

Across linear mixed‐effects models evaluating the additional volume extent on dMRI relative to CBCT, associations with clinical periodontal parameters varied by the dMRI sequence (Table 4). In the model including average PPD and number of BOP sites (*n* = 204 lesions), T1W additional lesion extent showed no significant reflection of either parameter (PPD: Estimate (*E*) = 0.0025, *p* = 0.252 and BOP: *E* = 0.0019, *p* = 0.132). Whereas, T1WC additional extent reflected average PPD only (*E* = 0.0058, *p* < 0.05). Meanwhile, T2W additional extent reflected both increasing PPD (*E* = 0.0344, *p* < 0.001) and number of BOP sites (*E* = 0.0087, *p* < 0.05).

**TABLE 4:** Association between periodontal inflammatory markers and additional lesion extent on dMRI.

Clinical parameters	Additional lesion volume extent on dMRI (dMRI – CBCT)					
	T1W		T1WC		T2W	
	Estimate	*p* ^a^	Estimate	*p* ^a^	Estimate	*p* ^a^
Average PPD	0.0025	0.252	0.0058	**< 0.05**	0.0344	**< 0.0001**
Number of BOP sites	0.0019	0.132	0.0019	0.181	0.0087	**< 0.05**
PISA	0.0003	**< 0.0001**	0.0002	**< 0.05**	0.0012	**< 0.0001**

*Note:* Bold values indicate statistically signfificant *p*‐values (*p* < 0.05).

Abbreviations: PPD, probing pocket depth; BOP, bleeding on probing; PISA, periodontal inflamed surface area; CBCT, cone‐beam computed tomography; T1W, T1‐weighted; T1WC, contrast‐enhanced T1‐weighted; T2W, T2‐weighted.

^a^ Statistical difference via linear mixed models (Kenward–Roger degrees of freedom).

PISA‐based models examined 168 lesions, as teeth with missing CAL and third molars were excluded. All dMRI sequences reflected PISA, but to differing degrees. T1W and T1WC additional extents showed significant but modest PISA effects as evidenced by estimates (*E* = 0.0003, *p* < 0.001 and *E* = 0.0002, *p* < 0.05, respectively). In contrast, the T2W additional extent demonstrated a larger effect and the strongest reflection of PISA (*E* = 0.0012, *p* < 0.001).

## 4 Discussion

This exploratory clinical pilot study evaluated the reliability of dMRI in detecting clinical signs of periodontal inflammatory status, relative to CBCT. First, radiation‐free dMRI showed diagnostic reliability comparable to that of CBCT for lesion detection and volumetrics. Second, lesion detectability and volumetric extent increased progressively with greater PPD and BOP, indicating that imaging mirrors clinical disease severity. Third, T2W identified the highest number of additional lesions, predominantly in negative and shallow PPD sites, highlighting potential for earlier disease recognition. Whereas CBCT and T1 weightings depicted periodontal bone loss to a comparable extent, T2W exhibited the strongest alignment with clinical inflammation indicators, notably PISA, underscoring its capability for estimating local inflammatory burden and potentially monitoring disease progression and therapy.

Methodological strengths include the use of dedicated dMRI coils and optimised 3D protocols, as well as a comprehensive in vivo blinded evaluation and comparison of multi‐contrast dMRI (T1W, T1WC, T2W) with the reference 3D imaging, namely CBCT. Imaging analyses paralleled clinical periodontal assessment at both site and tooth levels. Unlike earlier MRI studies, which restricted oedema quantification to molars (Probst et al. 2021; Schwarting et al. 2024), all tooth types were included. Importantly, periodontitis is a spatially complex 3D inflammatory process, whereas conventional clinical metrics such as PPD or BOP are linear, threshold‐based or simply dichotomous. Earlier CBCT and MRI studies therefore reduced imaging data to linear measurements for comparison with PPD, collapsing an inherently volumetric process into a 1D representation (Probst et al. 2021; Schwarting et al. 2024). Here, volumetric imaging changes were compared with BOP, closely reflecting biological behaviour complexity, while PISA was used as an intermediate bridging linear clinical measure and 3D imaging (Nesse et al. 2008; Park et al. 2017; Hujoel et al. 2001). Reliability results aligned with prior dMRI studies for detection (*κ* range = 0.884–0.987; this study 0.631–0.771) and volumetric analyses (ICC range = 0.78–0.91; this study 0.832–0.973) (Juerchott et al. 2020a, 2020b; Lauer et al. 2025), while no site‐level identification data is available for comparison.

Increasing PPD correlates positively with the degree of inflammation within the pocket (Zhong et al. 2007; Pradeep et al. 2009). Consistently, the number of lesions increased with greater PPD. While data on lesion detection rate versus PPD is lacking, prior research showed significant positive correlations between PPD and CBCT/T1WC/T2W lesion volumes (*r* range = 0.49–0.55), T2W oedema size (*r* range = 0.556–0.725), T1‐relaxation time (*r* = 0.957) and reduced T2W oedema depth accompanying reduced PPD after non‐surgical therapy (Probst et al. 2021; Schwarting et al. 2024; Lauer et al. 2025; Schara et al. 2009). Our results align with this but provided further findings. First, every dMRI contrast revealed more bone lesions than CBCT, suggesting higher sensitivity for disease recognition. Second, CBCT/T1W/T1WC exhibited similar lesion detection numbers at negative and shallow pockets, indicating limited sensitivity to early or moderately affected sites until structural changes become pronounced. In contrast, T2W identified substantially more lesions even at ≤ 3 mm PPD, commonly considered clinically healthy (Chapple et al. 2018), in agreement with reports of bone oedema at 2–3 mm PPD (Probst et al. 2021). All modalities also detected lesions even at 0–1 mm PPD sites, likely representing established bone loss in a reduced periodontium. T2W‐only lesions are, however, noteworthy. T2W′s high sensitivity or low specificity cannot be distinguished without histological ground truth. However, prior research has suggested that T2W can reveal bone oedema as a precursor to bone loss in gingivitis‐classified sites (Probst et al. 2021). In line with that, we propose that T2W‐only oedema without CBCT correlation may represent a transitional phase of early inflammation preceding bone loss in gingivitis on an intact periodontium or re‐activation of gingivitis on a reduced periodontium.

With regard to BOP, increasing bleeding was associated with more frequent lesion detection and larger volumes, reinforcing the link between inflammation severity and imaging‐based lesion expression. Previous studies reported T2W oedema size to be positively and significantly correlated to BOP (*r* = 0.264) and T2W oedema depth reduced with decreased bleeding following therapy (Probst et al. 2021; Schwarting et al. 2024). While CBCT/T1W/T1WC showed overlap to modest changes across bleeding categories, T2W exhibited a distinct bleeding‐volume relationship, with progressively larger volumes. T2W oedema volumes are inherently non‐specific and may be influenced by various physiological or pathological processes associated with increased marrow water content. However, the lack of meaningful associations in T1W sequences suggests that non‐specific signal variations alone are unlikely to fully account for the pronounced T2W expression. Instead, the selective and graded association between T2W lesion extent and bleeding severity suggests greater sensitivity to the biological process associated with periodontal inflammation. Nevertheless, this remains unproven with the available data and should be addressed in future studies.

Across bleeding levels, all dMRI lesion extents exceeded CBCT, suggesting MRI captures an additional marrow inflammatory component or is more sensitive for bone loss. Divergence, though, varied by sequence. T1W/T1WC volume increases closely paralleled CBCT, supporting the notion that T1 weightings primarily reflect structural changes, with contrast providing only incremental sensitivity. In contrast, T2W demonstrated disproportionate volume expansion with bleeding severity (*p* < 0.001). Previous studies have reported that T2W overestimates bone loss by 55% compared to CBCT (Lauer, et al. 2025) and 38%–89% compared to OPT (Probst et al. 2021). This study, however, nicely illustrates that bleeding severity is directly linked to oedema size, underlining T2W's sensitivity for periodontal detection. Recently, Lauer et al. identified a pattern where T2W volumes matched T1WC, corresponding to the CBCT‐defined bone loss. In the mismatch pattern, T2W signal abnormalities exceeded CBCT‐defined bone loss and T1WC signal changes and were most strongly associated with BOP, further supporting our findings (Laueret al. 2025).

Interestingly, only 20% of the additional lesion extent on T1W/T1WC was explained by bleeding, aligning with mixed‐effects models showing no meaningful BOP association. T1W showed no association with BOP or PPD, indicating that the additional extent likely reflects marginally overestimated bone lesion boundaries when CBCT is the reference. T1WC showed a low but significant association with PPD but none with bleeding, indicating that contrast agents may highlight limited perfusion or soft‐tissue changes around deeper pockets but remain insensitive to inflammation driving bleeding. In contrast, nearly 60% of T2W‐additional extent was explained by bleeding, with significant associations with both PPD and BOP. This consistent pattern proves T2W's ability to capture not only morphological changes evidenced by deeper pockets but also inflammatory‐induced and oedematous changes, characteristic of ongoing periodontal inflammation. PISA‐based models further reinforced this gradient across sequences. All dMRI sequences demonstrated a superior association with PISA compared to CBCT. T2W, however, displayed the strongest and most biologically coherent alignment, indicating that increases in inflamed epithelial surface area are mirrored by proportional increases in T2W lesion extent.

Taken together, these findings suggest that dMRI has the potential to depict periodontal bone loss with reliability comparable to CBCT, while also capturing inflammation‐related changes not detected by conventional radiography. Nevertheless, implementation in routine periodontal practice remains limited by higher costs, reduced availability and longer examination times. The absence of a priori sample size or power analysis represents an additional limitation. Therefore, these results should be interpreted as hypothesis‐generating and effect sizes used to inform future studies. The smaller number of deep‐pocket sites may reduce the robustness of conclusions regarding advanced local disease. This, however, reflects the site‐specific nature of periodontitis, as deep pockets affect only a limited number of sites even in patients with severe disease. Pooling stage III and IV and grades B and C patients may also obscure stage‐ and grade‐specific imaging patterns. Artefact exclusion rates were intermediate to the 4.6%–15% previously reported (Probst et al. 2021; Schwarting et al. 2024; Lauer et al. 2025), mainly present in T1W sequences (tooth and site levels: 7.0% and 8.3% in T1W vs. 2.5% and 2.3% in T2W). Diagnostic accuracy and specificity of dMRI remain to be elucidated; however, histological reference in clinically healthy sites is an ethical issue. Recent reports also showed that T1W perfusion imaging demonstrated superior sensitivity for assessing lesion extent even compared to T2W and a significant relation to BOP (Lauer et al. 2025), enabling separation of bone loss from periodontal oedema. However, as no data on lesion identification sensitivity or treatment response evaluation are available, its value remains to be elucidated in future studies.

## 5 Conclusion

In this in vivo periodontitis study, dMRI showed reliability equivalent to CBCT for lesion identification and volume quantification. Both CBCT and T1W depicted periodontal bone loss to a comparable extent. T2W stood out as the contrast identifying the highest number of periodontal lesions and demonstrating the strongest association with PPD, BOP and PISA, highlighting its potential for early disease recognition and assessment of inflammatory burden. As no added value of intravenous contrast‐enhanced T1WC was noted, T1W and T2W contrasts seem promising and sufficient for assessing periodontal bone loss and inflammation.

## Author Contributions

N.T., T.H., A.L., S.H. and M.B. contributed to the conceptualisation and design of the study. N.T., T.H. and A.L. contributed to the methodology, investigation and formal analysis. N.T., M.K. and H.L. conducted the statistical analyses and contributed to the interpretation of the statistical results. K.H., L.S., A.J., M.S., C.H., M.R., M.N., S.H. and M.B. contributed to data curation, resources and critical review and editing of the manuscript. T.H. led project administration and funding acquisition. A.J. also contributed to funding acquisition. N.T. and T.H. drafted the original manuscript. All authors have read and approved the final version of the manuscript and agree to be accountable for all aspects of the work.

## Funding

This work was supported by Dietmar Hopp Stiftung, 1DH2011152.

## Conflicts of Interest

Dr. Hilgenfeld and Dr. Juerchott report receiving grants from Dietmar Hopp Foundation during the conduct of the study. Dr. Nittka is an employee of Siemens Healthineers AG, Erlangen, Germany.

## Supporting information


**Table S1:** Sequence parameters used for dental magnetic resonance imaging.
**Table S2:** Site‐specific lesion detection and tooth‐specific lesion volumetrics intra‐ and inter‐rater reliability of CBCT and dMRI.
**Table S3:** Periodontal lesion detection at singles sites according to combined probing depth categories (PPD ≤ 3 vs. > 3 mm).
**Table S4:** BOP category pairwise comparisons of T2W and T2W‐CBCT periodontal lesion volumes.

**Appendix A:** Extended Methodological and Statistical Details.
**Appendix B:** Extended Results for Lesion Detection and Volumetric Analyses.

## Data Availability

The data that support the findings of this study are available on request from the corresponding author. The data are not publicly available due to privacy or ethical restrictions.
