# Supplementary material for: A Pilot Comparative Study of Multi‐Contrast Dental MRI and CBCT: Clinical Correlates of Disease Status and Inflammatory Burden in Periodontitis
Source: J Clin Periodontol. 2026 Jun 17;53(9):1382–92. doi: 10.1111/jcpe.70151 (PMC13477842; doi:10.1111/jcpe.70151)
Supplement: Supplementary file 1 [file JCPE-53-1382-s001.pdf]

Appendix A

Extended Methodological and Statistical Details

Study Participants

Patients were recruited from an ongoing periodontitis cohort study between September 2017 and December 2022 at the University Hospital Heidelberg. Inclusion criteria comprised diagnosis of advanced periodontitis (stage III, grade B/C or higher), no periodontal therapy within the preceding 3 month and the availability of complete clinical examinations together with CBCT.

Image Processing and Periodontal Lesion Analysis

Imaging presentation of periodontal lesion was defined as follows: For CBCT, osseous defects consistent with periodontitis, including an increased cemento-enamel junction to alveolar crest distance > 2 mm and/or a visible infrabony or inter-radicular defect morphology. For MRI, tooth-associated intraosseous signal changes extending from the supposed level of the alveolar ridge to the deepest extent of signal abnormalities and clearly distinguishable from normal bone marrow signal (Figure 1).

Tooth-Specific Analysis: Periodontal Lesion Volumetrics

Segmentation was performed manually using 3D-SLICER on each slice, and visualisation parameters (such windowing and threshold adjustment) were adapted on a lesion-by-lesion basis to best delineate lesion borders. Outlined volumes were calculated, and the median volume was used for statistical analysis.

Reliability Analysis of Image Assessments

Readings were performed by four observers (N.T: dentist and oral radiologist with 8 years of experience in dental imaging; A.L., L.S. and K.H.: neuroradiologists with 10, 4 and 5 years of experience in dental imaging). For an analysis type (qualitative or quantitative) and an image modality/sequence, one dedicated reader assessed the complete dataset to avoid variability introduced by multiple readers within the modality/sequence. For intra-reader reliability, the same first reader re-evaluated 25% of the data, which was randomly chosen, as per previous studies (Feuerriegel et al. 2023; Madi et al. 2024; Fontenele et al. 2022), and for inter-reader reliability, a second reader re-evaluated 25% of the data for the third time. To ensure consistency across readers and over time, all readers underwent training sessions prior to each formal analysis, during which the criteria for lesion definition, detection and boundary delineation were agreed upon.

Statistical Analysis

Statistical analyses were performed using IBM SPSS Statistics Version 29 (IBM Corp, Armonk, NY), MATLAB R2024b or SAS version 9.4 (SAS Institute Inc). Intra- and inter-rater reliability of site- and tooth-specific analyses were evaluated by Cohen's kappa (κ) and intra-class correlation coefficient (ICC), and interpreted using agreement descriptors proposed by Landis and Koch (Landis and Koch 1977a, 1977b). Reliability for volumetric measurements was assessed using a two-way mixed-effect model with absolute agreement for single measurements (ICC [3,1]). Normal distribution was assessed using the Shapiro–Wilk test. Volumes and differences between dMRI and CBCT across BOP categories were tested using the Kruskal–Wallis test with Bonferroni's correction for pairwise comparison. The relationship between additional dMRI extent and the number of bleeding sites was evaluated using exponential regression models, and goodness of fit was tested using nonlinear least-squares estimation in MATLAB R2024b with default convergence criteria. Model fit was evaluated using R^2. Linear mixed models (Kenward–Roger degrees of freedom) were used to assess the relationship between additional extent and clinical parameters (average PPD, number of BOP sites, PISA), with CBCT volumes as a covariate using the SAS software. A random intercept for patient was included to define the covariance structure and to account for within patient correlation (several teeth per patient). Missing data were handled by fitting each model using all available data for the clinical parameter included in that model. Two-tailed p-values < 0.05 were considered statistically significant.

Appendix B

Extended Results for Lesion Detection and Volumetric Analyses

Association Between PPD and Lesion Detection

Sites were distributed as follows: Negative PPDs = 1168, shallow PPDs = 752 and deep PPDs = 66 sites (Table 1). Overall, CBCT/T1W/T1WC/T2W identified 1038/1163/1262/1592 sites with periodontal lesions, respectively.

Association Between BOP Sites and Lesion Volumes

Teeth were distributed as follows: $BOP^{0} = 89$, $BOP^{1+} = 70$, $BOP^{2+} = 44$, $BOP^{3+} = 56$, $BOP^{4+} = 40$, $BOP^{5+} = 10$ and $BOP^{6+} = 33$ teeth (Table 2). Overall, 204 periodontal lesions were identified, and detectability increased with number of BOP sites: $BOP^{0/1+/2+/3+/4+/5+/6+}$: 30 (33.7%)/41 (58.6%)/30 (75.0%)/37 (66.1%)/34 (85.0%)/8 (80.0%)/24 (72.7%).
