# Supplementary material for: A Pilot Comparative Study of Multi‐Contrast Dental MRI and CBCT: Clinical Correlates of Disease Status and Inflammatory Burden in Periodontitis
Source: J Clin Periodontol. 2026 Jun 17;53(9):1382–92. doi: 10.1111/jcpe.70151 (PMC13477842; doi:10.1111/jcpe.70151)
Supplement: Supplementary file 2 [file JCPE-53-1382-s002.docx]

Supplementary Table S1. Sequence parameters used for dental magnetic resonance imaging.

|  | T2W MSVAT-SPACE | 3D T1W VIBE with Dixon-based fat suppression |
| --- | --- | --- |
| Voxel size (mm^3^) | 0.6 × 0.6 × 0.6 | 0.7 × 0.7 × 0.7 |
| Echo time (ms) | 236 | 2.45 |
| Repetition time (ms) | 2500 | 15.6 |
| Inversion time (ms) | 200 | – |
| Flip angle (°) | 120 | 14.9 |
| Field of view (mm^2^) | 191 × 151 | 223 × 153 |
| Receiver bandwidth (Hz/pixel) | 521 | 601 |
| Acquisition matrix | 320 × 252 | 220 × 320 |
| Number of sections | 104 | 80 |
| Acquisition time (s) | 425 | 362 |

Abbreviations: T2W:  T2-weighted, MSVAT: Multislab acquisition with view-angle tilting gradient, SPACE: Sampling perfection with application-optimized contrasts using different flip-angle evolution, T1W: T1-weighted, VIBE: Volumetric interpolated breath-hold examination

Supplementary Table S2. Site-specific lesion detection and tooth-specific lesion volumetrics intra- and inter-rater reliability of CBCT and dMRI

|  | | CBCT | T1W | T1WC | T2W |
| --- | --- | --- | --- | --- | --- |
| Site-specific lesion detection | |  |  |  |  |
|  | Intra-rater (κ) | 0.751  [0.701-0.800] | 0.707  [0.654-0.761] | 0.703  [0.649-0.757] | 0.771  [0.710-0.833] |
|  | Inter-rater (κ) | 0.736  [0.685-0.787] | 0.682  [0.627-0.737] | 0.689  [0.633-0.744] | 0.631  [0.560-0.702] |
| Tooth-specific lesion volumetrics | |  |  |  |  |
|  | Intra-rater (ICC) | 0.885  [ 0.786-0.935] | 0.928  [0.890-0.953] | 0.936  [0.878-0.963] | 0.904  [0.847-0.940] |
|  | Inter-rater (ICC) | 0.917  [0.864-0.949] | 0.907  [0.832-0.945] | 0.973  [0.958-0.983] | 0.832  [0.734-0.894] |

Abbreviations: κ: Cohen’s kappa, ICC: Intraclass-correlation-coefficient, CBCT: cone-beam computed tomography, T1W: T1-weighted, T1WC: contrast-enhanced T1-weighted, T2W: T2-weighted.

Supplementary Table S3. Periodontal lesion detection at singles sites according to combined probing depth categories (PPD≤ 3mm vs. > 3mm)

|  | PPD≤ 3mm (n=1920) | PPD> 3mm (n=66) | Relative increase lesion detection (%) |
| --- | --- | --- | --- |
| CBCT, n (%) | 983 (51.2) | 55 (83.3) | 62.8 |
|  |  |  |  |
| T1W, n (%) | 1104 (57.5) | 59 (89.4) | 55.5 |
| T1WI % compared to CBCT (%) | 12.3 | 7.3 |  |
|  |  |  |  |
| T1WC+, n (%) | 1202 (62.6) | 60 (90.9) | 45.2 |
| T1WC+ % compared to CBCT (%) | 22.3 | 9.1 |  |
|  |  |  |  |
| T2W, n (%) | 1529 (79.6) | 63 (95.5) | 19.9 |
| T2W % compared to CBCT (%) | 55.5 | 14.5 |  |

Abbreviations: PPD: probing pocket depth, CBCT: cone-beam computed tomography, T1W: T1-weighted, T1WC: contrast-enhanced T1-weighted, T2W: T2-weighted.

Supplementary table S4. BOP-category pairwise comparisons of T2W and T2W-CBCT periodontal lesion volumes

|  | T2W | | T2W-CBCT | |
| --- | --- | --- | --- | --- |
|  | p-value | Adjusted p-value* | p-value | Adjusted p-value* |
| BOP^0^ vs. BOP^1+^ | 0.586 | 1.000 | 0.806 | 1.000 |
| BOP^0^ vs. BOP^2+^ | 0.007 | 0.141 | 0.060 | 1.000 |
| BOP^0^ vs. BOP^3+^ | 0.006 | 0.125 | 0.066 | 1.000 |
| BOP^0^ vs. BOP^4+^ | 0.068 | 1.000 | 0.156 | 1.000 |
| BOP^0^ vs. BOP^5+^ | 0.069 | 1.000 | 0.101 | 1.000 |
| BOP^0^ vs. BOP^6+^ | 0.000 | **0.000** | 0.000 | **0.001** |
| BOP^1+^ vs. BOP^2+^ | 0.018 | 0.376 | 0.076 | 1.000 |
| BOP^1+^ vs. BOP^3+^ | 0.016 | 0.343 | 0.083 | 1.000 |
| BOP^1+^ vs. BOP^4+^ | 0.159 | 1.000 | 0.202 | 1.000 |
| BOP^1+^ vs. BOP^5+^ | 0.125 | 1.000 | 0.125 | 1.000 |
| BOP^1+^ vs. BOP^6+^ | 0.000 | **0.000** | 0.000 | **0.001** |
| BOP^2+^ vs. BOP^3+^ | 0.921 | 1.000 | 0.891 | 1.000 |
| BOP^2+^ vs. BOP^4+^ | 0.333 | 1.000 | 0.602 | 1.000 |
| BOP^2+^ vs. BOP^5+^ | 0.952 | 1.000 | 0.675 | 1.000 |
| BOP^2+^ vs. BOP^6+^ | 0.052 | 1.000 | 0.025 | 0.522 |
| BOP^3+^ vs. BOP^4+^ | 0.358 | 1.000 | 0.683 | 1.000 |
| BOP^3+^ vs. BOP^5+^ | 0.902 | 1.000 | 0.607 | 1.000 |
| BOP^3+^ vs. BOP^6+^ | 0.034 | 0.705 | 0.013 | 0.282 |
| BOP^4+^ vs. BOP^5+^ | 0.498 | 1.000 | 0.449 | 1.000 |
| BOP^4+^ vs. BOP^6+^ | 0.004 | 0.076 | 0.005 | 0.109 |
| BOP^5+^ vs. BOP^6+^ | 0.213 | 1.000 | 0.273 | 1.000 |

*P-value adjusted by the Bonferroni correction for multiple tests.
